# Artificial Intelligence Tools in Precision Lung Cancer Care: From Early Detection to Clinical Decision Support

**DOI:** 10.3390/cancers18091455

**Published:** 2026-05-01

**Authors:** Christopher R. Grant, Sandip P. Patel, Tali Azenkot

**Affiliations:** San Diego Moores Cancer Center, University of California, La Jolla, CA 92037, USA; spatel@health.ucsd.edu (S.P.P.); tali.azen@gmail.com (T.A.)

**Keywords:** artificial intelligence, machine learning, deep learning, precision medicine, thoracic oncology, lung cancer

## Abstract

Thoracic malignancies are uniquely positioned for the integration of emerging technologies such as artificial intelligence (AI), which have the potential to advance precision oncology across the cancer care continuum. In cancer screening, AI has emerged as a promising strategy to enhance diagnostic accuracy, efficiency, and scalability. Deep learning applied to pathology (pathomics) and imaging (radiomics) has enabled the development of novel, noninvasive tools capable of predicting histologic and molecular features that may correlate with treatment response or toxicity. In drug discovery, computational approaches can analyze large-scale genomic, chemical, and clinical datasets to accelerate target identification and match candidate compounds to available targets; this may be particularly useful in the context of resistance to targeted therapy. AI tools may also support treatment planning for radiation and surgery, guide systemic therapy selection, and facilitate continuous monitoring for early identification of treatment resistance or toxicity. As these technologies are integrated into clinical workflows, careful attention to ethical, regulatory, and clinical governance frameworks will be essential to ensure equitable implementation and bias mitigation. Maintaining human oversight and a human-centered approach remain critical, as complex treatment decisions and sensitive patient interactions are central to the care of patients with thoracic malignancies.

## Simple Summary

Artificial intelligence (AI), the field of computer science that builds systems to simulate human intelligence, is increasingly applied in oncology. In lung cancer, AI-based tools offer a unique opportunity to advanced precision oncology given the central role of genetic testing and imaging for diagnosis, treatment selection, and disease monitoring. In this review, we summarize the potential role for AI interventions across the lung cancer care continuum. We discuss established and emerging applications, including screening scan interpretation, tumor biomarker prediction from pathology or imaging data, support for surgical or radiation treatment planning, and detection of treatment-related side effects, such as through wearable technologies. We highlight concerns about algorithmic bias and outline strategies to mitigate these risks. We also examine key ethical and regulatory considerations surrounding the integration of AI into clinical practice. Overall, AI tools may advance lung cancer care by enabling more personalized clinical decision-making. The development of clear validation standards, equitable implementation, and ongoing human oversight are essential to the safe and effective adoption of new technologies.

## 1. Introduction

Lung cancer remains the leading cause of cancer mortality worldwide, accounting for an estimated 1.8 million deaths globally, or 18.7% of total cancer deaths, in 2022 [1]. Despite advances in screening and therapeutics, outcomes remain poor for many patients, underscoring the need for improved strategies in early detection, treatment selection, and toxicity mitigation. Thoracic malignancies are particularly well suited for the integration of emerging technologies that support precision oncology, an approach that aims to tailor treatment according to patient-specific histologic, molecular, and clinical characteristics [2].

Several features of lung cancer make it an ideal setting for technological innovation. First, its high global prevalence has generated large clinical, imaging, and molecular datasets available for computational analysis and algorithm development. Second, the central role of imaging modalities, including computed tomography (CT) scans for population-based screening and disease monitoring, provides abundant data for automated interpretation and decision support. Finally, the substantial molecular heterogeneity of non-small cell lung cancer (NSCLC), together with a rapidly expanding landscape of targeted therapies, creates opportunities to better predict and optimize clinical outcomes.

Artificial intelligence (AI) represents one of the most promising technological advances applied to oncology. AI broadly refers to machine-based systems capable of analyzing input data to generate predictions, recommendations, or decisions (Table 1) [3]. Within this framework, machine learning algorithms ascertain patterns from data to generate predictive models, while deep learning uses neural networks to process and identify hypothesis-generating patterns in large datasets [3]. Improvements in computational power, data availability, and analytic methods have accelerated the development of increasingly sophisticated AI applications across cancer care research and clinical care [4,5].

AI-enabled technologies are already being incorporated into oncology practice. Patients and clinicians increasingly interact with large language models (LLMs), generative AI systems capable of producing human-like responses to complex clinical queries using natural language inputs [6]. At the same time, health systems are integrating computer-aided detection (CADe) and computer-aided diagnosis (CADx) tools, which leverage deep learning algorithms to assist with interpretation of pathology and imaging data [7]. For each intervention, identifying the intended context of use is central to assessing its potential contribution to patient care.

CADe and CADx tools must undergo rigorous technical and internal validation, external validation with independent datasets, comparison to the current standard of care, and prospective validation to achieve regulatory approval. In the United States, AI-based software (e.g., for lung nodule detection) are regulated as medical devices by the Food and Drug Administration (FDA) [8]. Most CADe tools are evaluated for regulatory approval via the FDA’s 510(k) pathway, which requires demonstration of “substantial equivalence” to a device that is already legally marketed [9]. Currently, Eyonis Lung Cancer Screening, RevealDx, Optellum Virtual Nodule Clinic, qCT LN Quant, and VisRad XR are examples of FDA-approved AI-based lung cancer screening tools [10,11,12,13,14].

Key features that distinguish AI technologies are their degree of explainability (the ability to understand mechanisms underlying AI system operation) and interpretability (the ability to understand the meaning of the AI system output) [3]. In clinical care, interpretability is not optional, as clinicians must provide transparent justification for decisions that affect their patients [15]. For some clinical scenarios, more transparent approaches (consider statistical models, such as logistic regression) might be preferable to an AI system due to greater explainability, even in the face of potentially lower predictive accuracy [16].

In this review, we examine recent advances in the application of AI across the continuum of lung cancer care, from population-level screening and early detection to individualized treatment selection and disease monitoring (Table 2). Across these applications, careful attention to ethical, regulatory, and clinical governance frameworks remains essential. The responsible integration of AI must ensure transparency, equity, and accountability so that these technologies enhance, rather than exacerbate, disparities in cancer care [17]. As has been noted in the field of radiology, AI is unlikely to replace clinicians; rather, the integration of AI into clinical workflows may augment clinician capabilities and, as we argue for lung cancer, advance personalized care [18].

## 2. AI in Precision Lung Cancer Diagnostics

### 2.1. Lung Cancer Screening

Early detection of lung cancer substantially improves survival and remains one of the most impactful strategies for reducing lung-cancer-related mortality [19,20]. As a result, the integration of AI into lung cancer screening workflows has emerged as a promising approach to enhance diagnostic accuracy, efficiency, and scalability of screening protocols [21,22]. Large, well-annotated imaging datasets have facilitated the development and validation of these approaches. Imaging data generated through landmark screening trials, including the National Lung Screening Trial in the United States and the Pan-Canadian Early Detection of Lung Cancer Study (PanCan), have provided valuable resources for training and validating novel algorithms. The International Association for the Study of Lung Cancer (IASLC) also sponsored development of the Early Lung Imaging Confederation (ELIC) resource in 2018, which is an open-source screening imaging dataset compliant with international privacy regulations [23].

AI models have been applied across multiple aspects of lung cancer screening workflows. Machine learning approaches may improve identification of individuals most likely to benefit from screening [24], evaluate screening images before or alongside a radiologist [25,26], or perform a risk assessment of identified lung nodules [27,28]. One clinically validated deep learning model, Sybil, was trained using low-dose CT scans from the National Lung Screening Trial and then was validated on three large independent datasets as follows: 6282 CTs from the National Lung Screening Trial, 8821 CTs from Massachusettes General Hospital, and 12,280 CTs from Chang Gung Memorial Hospital. Sybil demonstrated the ability to predict future risk of lung cancer development from a single low-dose CT scan [29]. While promising, Sybil has several vulnerabilities in its processing, such as the ability to correctly lateralize the location of future cancers, focusing on specific at-risk regions based on its training data rather than equally across the entire thorax. Additionally, Sybil had a lower rate of future cancer risk when lung nodules already identified as cancerous were removed from the dataset. If trained on a specific dataset, deep learning models have the potential to aid unmet clinical needs, including identifying never-smoker, high-risk populations who could harbor NSCLC with actionable genomic alterations; notably, this cohort is excluded from lung cancer screening protocols in most settings.

Another more exploratory algorithm called CXR-Lung-Risk focused on predicting lung cancer mortality from a chest radiograph [30]. While this has a potential for clinical utility, it is unknown which chest radiograph findings are important for the prediction of lung cancer. The model’s training dataset was based on imaging from mostly non-hispanic, white patients, which risks extrapolation to other patient populations that may present with different radiographic findings. Further training on diverse datasets and external validation among heterogeneous populations is warranted to confirm clinical applicability.

Prospective evidence supporting the clinical implementation of AI-based CADe programs is also emerging. In an early randomized trial, an AI-based CAD-implemented software, coined Lunit INSIGHT CXR version 2.0.2.0, assessed participants undergoing chest radiograph screening and randomized them to AI-assisted or standard interpretation [31]. AI assistance significantly improved detection of actionable and malignant lung nodules, with similar false-positive report rates among AI-assisted and standard of care interpretation. This study is a laudabile randomized controlled trial; however, limitations include that not all participants underwent CT imaging, and the diagnostic chest radiography group was not powered to detect clinical significance. Additionally, this is a single-institution study and the estimated sample size for the trial was not achieved. Clinical expertise from a multi-insitutional prospective trial is needed to validate this and other models prior to implementation. Additional prospective studies are ongoing, including trials evaluating the impact of AI-assisted screeening on health system resource utlization and investigations targeting specific populations, such as Asian never-smokers who are excluded based on current screening criteria [32,33].

Despite these advances, important questions remain regarding the optimal regulation and integration of AI in lung cancer screening programs. As discussed in the introduction, AI-based software is regulated as medical devices and most commonly approved through the FDA’s 510(k) pathway in the United States [8]. Common endpoints for model performance include receiver operating chracteristic (ROC) curve metrics, primarily area under the curve (AUC), as well as sensitivity and specificity [9]. After regulatory approval, challenges remain to real-world integration of screening technologies. These include defining the most appropriate use cases and target populations for AI deployment, ensuring the generalizability of models developed in specific datasets to broader populations, and addressing medicolegal considerations around clinical implementation. Continued prospective validation and careful integration into existing screening frameworks will be essential to ensure that AI enhances early detection strategies, as well as other aspects of clinical care.

### 2.2. Pathomics and Digital Pathology

While imaging is central to the initial detection of lung cancer, definitive diagnosis and therapeutic decision-making rely on tissue evaluation. Advances in digital pathology, such as the digitization of whole slides, have facilitated the application of AI to histopathologic images. The field of pathomics refers to the quantitative analysis of features from digitized slides to capture sub-visual characteristics that reflect tumor biology and behavior [34]. Through extraction of high-dimensional data, pathomics enables the computational analysis of tissue architecture, cellular morphology, and tumor microenvironment patterns that may not be discernible to the human eye.

One deep learning algorithm, named NAVF-bio, was trained on a retrospective dataset of 741 haematoxylin- and eosin-stained lung tissue slides with established diagnoses [35]. NAVF-bio had a strong performance in distinguishing malignant tumors from benign processes and in determining tumor histology (e.g., adenocarcinoma versus squamous carcinoma) when compared to four pathologists of different professional levels (senior attending, junior attending, and trainee) [35]. The model performance was limited for small slides, slides with fewer tumor cells, and obvious artifacts such as pen marks, margin overlap, and defocus. Further external prospective validation is required for NAVF-bio and similar modes prior to real-world application.

AI tools also have potential to automate interpretation of established biomarkers, such as quantifying programmed death-ligand 1 (PD-L1) expression. Lunit SCOPE PD-L1 was initially evaluated using 4675 immunohistochemistry stained 0.04 mm^2^ tissue grids from 802 patients with NSCLC. There was distinct concordance with the pathologist in PD-L1 immunohistochemistry scoring retrospectivly for tumor proportion score (TPS) ≥50% (85.7%), TPS 1–49% (89.3%), and TPS <1% (52.4%). While encouraging, only cases with at least 20 tumor cells per patch that were whole slide images were included. Additionally, there was decreased concordance for TPS <1% that could suggest limited accuracy on these samples. Lunit SCOPE PD-L1 was trained on 22C3 assays, excluding other clones such as SP263, 28-8, and SP142. Given the geographic and institutional variability of assays, this model would largely be restricted to centers that use 22C3 and require further analysis on other assays. 

AI tools may also allow for the implementatioan of more sophisticated immunohistochemistry interpretation. For example, the normalized membrane ratio-quantitative continuous scoring (NMR-QCS) identifies surface-to-cytoplasm ratios of target protein expression. NMR-QCS for trophoblast cell surface antigen 2 (TROP2) is the first AI-based companion diagnostic test, approved for the antibody–drug conjugate datopotamab deruxtecan in patients with NSCLC [36]. Interpretation of other immunohistochemistry markers, such as Human Epidermal Growth Factor Receptor 2 (HER2) expression, is an area of need, though heterogeneity of staining in combination with low cutoff values may provide a challenge in developing AI models.

In addition to histologic features, deep learning algorithms have been deployed to predict molecular alterations. Several studies using whole slides from The Cancer Genome Atlas (TCGA) and institutional sources have demonstrated successful discrimination of *TP53*, *EGFR*, *ALK*, *KRAS*, *STK11*, and other mutations, suggesting that morphological features may correlate with underlying genomic alterations [37,38,39]. This has yet to be compared to traditional molecular testing and requires external validation. More recently, NAVF-Bio has also been explored in its integration of histologic features with spatial transcriptions and tumor microenvironment characterization to predict multiple driver gene mutations and TMB status on three external datasets [40]. NAVF-Bio showed a precision rate ranging from 77% to 83% on the external datasets, with potential for a high false positive rate that may lead to sinister clinical consequences in real world settings.

Beyond diagnostics, AI-assisted digital pathology is also being investigated to predict treatment benefit. For example, one exploratory model used in predicting pathologic response for those treated with neoadjuvant atezolizumab had strong concordance with pathologists in determining viable tumor percentage (87%) and predicting major pathologic response of <10% viable tumor (92.1%) [41]. Tools such as this may increase speed of pathologic assessment and/or aid pathologists in reviewing difficult cases as a second opinion. There is also a potential for the development of algorithms that may analyze interactions between tumor cells, immune infiltrates, and stromal components as predictive biomarker signatures that are otherwise illegible to the human eye [42]. As digital pathology platforms continue to evolve, diagnostic and predictive insights that support precision treatment selection for patients with lung cancer could be developed after rigorous prospective analysis.

In this and other AI-driven fields, there remains a significant need for standardized metrics to evaluate model performance over time. To date, relatively few studies have undertaken systematic, multi-model comparions of computational pathology approaches to biomarker assessment. Innovative collaborative efforts, including those led by the not-for-profit Friends of Cancer Research, have begun to address this gap by establishing benchmarking strategies such as for AI-enabled quantification of human epidermal growth factor receptor 2 (HER2) expression by immunohistochemistry in breast cancer [43]. Similar approaches will benefit standardization of AI-enabled biomarker assessment in thoracic malignancies such as NSCLC, in which up to 50% of patients harbor actionable genomic mutations [44].

### 2.3. Radiomics

Like the inference of molecular features from histopathology slides, advances in imaging analysis have raised the possibility that some tumor characteristics may be determined without confirmatory tissue sampling. Radiomics, the analysis of quantitative features from imaging modalities, translates images to minable data that has the potential to function as biomarkers for diagnosis, prognostication, and treatment monitoring [45]. Radiomic algorithms have been applied across the spectrum of cancer care, including diagnosis, staging, and treatment response or toxicity prediction. The Cancer Imaging Archive managed by the National Cancer Institute (NCI), is one public repository of imaging data that has been used for radiomics research; it includes several collections dedicated to chest imaging [46].

In lung cancer, a growing body of literature explores the correlation between radiomic features and tumor biology. Radiomic models have been investigated to predict driver mutations, PD-L1 expression levels, and tumor mutation burden, postulating that imaging may serve as a noninvasive, “virtual biopsy” surrogate for molecular testing [45,47]. Considerable effort has focused on predicting *EGFR* mutation status in NSCLC, with studies using CT imaging and magnetic resonance imaging (MRI) features showing promising accuracy [48,49]. These “virtual biopsies” may be particularly relevant in settings where tissue is limited or not yet available, such as in brain metastases or pre-operative assessment [50,51].

Beyond molecular prediction, radiomics has also showed promise in supporting clinical decision-making in lung cancer. Radiomic models derived from CT and positron emission tomography (PET)/CT imaging have demonstrated utility in predicting lymph node metastasis and improving nodal staging accuracy in NSCLC [52]. Radiomic signatures have been investigated to predict response to chemoimmunotherapy in the neoadjuvant setting and immune checkpoint inhibitiors in advanced disease [53,54,55]. This is very important, as several favorable surrogate endpoints, such as pathologic complete response and major pathologic response, show improved overall survival in the neoadjuvant and perioperative immunotherapy era. Incorporating radiomics has the potential to help tailor neoadjuvant duration of treatment to best optimize patient outcomes while limiting treatment-related toxicity.

Radiomics has further been explored for toxicity prediction. For example, one algorithm incorporating both CT-based radiomic features and a deep learning recognition algorithm had a sentitivity of 77.6% and a positive predictive value of 25.4% for identifying patients at risk of immune checkpoint inhibitor-associated pneumonitis [56]. While the postitive predictive value is evidence that deep learning models are not close to the level of human detection, future generations of models may build on this intervention.

As discussed for lung cancer screening programs, well-defined regulatory pathways are necessary for clinical implementation of radiomic models. While the majority of FDA-approved AI software devices are in the field of radiology, over 20% of these devices focus on chest radiology [8,57]. Capabilities include lung nodule detection, emergent changes identification (e.g., pneumothorax or pulmonary embolism), and indwelling catheter monitoring [57]. The high volume of thoracic device approval highlights the potential of AI to improve thoracic malignancy care.

Several challenges to the application of radiomics in the treatment of lung cancer remain, limiting radiomic platform use in clinical practice. For example, many studies lack both standardized evaluation metrics and external validation. This is compounded by the fact that several of the studies highighted here are trained on publically available datasets such as those from Cancer Imaging Archive, Stanford Radiogenomic NSCLC dataset, or NSCLC-Radiomics. While these datasets provide useful training imaging, their small sample size may blunt a language learning model’s development. One important bias to consider in predictive models such as these is overfitting, the scenario where a model is trained on specific data to the extent that it does not generalize well to new data [16]. For example, in NSCLC, a model that predicts *EGFR* mutations may not demonstrate the same sensitivity or specificity when applied from Asian to Western populations, given differences between the two groups [44]. Until models are trained and applied to specific patient populations, standardized reporting is instituted, and external validation guidelines are utilized, applicability of radiomics to tumor biopsy will remain in the experimental phase.

## 3. AI in Precision Lung Cancer Therapeutics

### 3.1. Drug Discovery

AI-based technologies have also demonstrated promise in refining the oncology drug discovery pipeline. Translational drug discovery is a resource-intensive process, and computational approaches have the potential to analyze large-scale genomic, chemical, and clinical datasets to accelerate target identification and compound-target matching. Various machine learning models are under evaluation in lung cancer for drug discovery. Here we highlight three specific techniques that have shown promise to potentially change clinical practice.

First, random forest classifier is a machine learning model that can analyze genetic variants and lung cancer suceptibility factors (e.g., structural varients, gene expression profiles, DNA methylation patterns, and miRNA signatures) to identify biomarkers of treatment response and assess patient progonsis [58]. It functions as a collection of decision trees that make multiple predictions by splitting data based on various features, such as tumor size, tumor location, and demographics. Uniquely, this method has a cross-validation techinique with different sample sizes to improve the model with each iteration, avoiding overfitting. One study using the KOBAS gene oncology index showed that a random forest classifer had an 87% accuracy in predicting biomarkers in lung cancer, outperforming other competitor machine learning models [59]. However, this model uses various training sample sizes that predispose it to sample bias. It is also less interpretable than a single-decision tree when the consensus output has a narrow margin. This model highlights the need for well-defined parameters in random forest classifiers, as well as across all machine learning models.

Second, gradient boosting is a promising techqniue, where predictions are made by using simple models where each new model tries to correct the mistakes of the previous ones sequentially. This technqiue is uniquely positioned to aid scientists’ understanding of overlapping gene signatures and future resistance mechanisms. Each new prediction learns from prior errors of the last prediction when training, and thus can learn from specific bypass mechanisms. Studies using gradient boosting have been completed in screening radiomics with a high level of accuracy (92.98%), true positive rate (94.98%), and true negative rate (90.87%) using a retrospective lung imaging database [60]. One study demonstrated how gradient boosting can predict Wnt pathway overlapping genes in all cancers with a 78.9% accuracy [61]. This type of machine learning positions well in precision medicine for NSCLC (wherein serial biopsies are often obtained), as it may detect novel molecular targets and resistance mechanisms. This technique, however, may lead to overfitting since correlations are performed sequentially, creating poor external validation results due to random fluctuations in learned training data. Future generations of gradient boosting models may be able to overcome overfitting with the use of more training data and a simplified model with decreased depth to avoid the inherent flaws of the deep neural complexes.

Lastly, deep neural networks is a type of learning model that may also leverage comprehensive target profiles to inform drug repurposing strategies, thereby expanding the therapeutic utility of existing drugs [62]. Deep neural networks consist of hidden layers that extract more abstract features to provide a precise output. Most recently, one model, DeepSynergy, has been used to determine specific personalized drug combinations that would maximize drug synergy [63]. In one study, DeepSynergy demonstrated an AUC of 0.90, which was better than its comparators such as gradient boosting machines (AUC 0.89) and random forests (AUC 0.87) [63]. Notably, deep learning networks require a plethora of data for training; else, they risk overfitting due to the inherent in-depth processing.

To complement and train several of the drug discovery learning models, a growing number of publicly available databases have been created to facilitate AI-driven drug discovery [64]. The Cancer Distributed Learning Environment (CANDLE) is one open-source software platform sponsored through the Exascale Computing Project that uses a deep neural network with the intention to train large numbers of computational models and predict drug interactions with various cancer types [65]. The Predictive Oncology Model and Data Clearinghouse (MoDaC) is an additional data repository of predictive oncology datasets that can be used to validate learning models. While not formally tested, both CANDLE and MoDaC have small sample sizes that lack heterogeneity, which will hopefully be improved upon in future datasets.

### 3.2. Clinical Decision and Prognostic Tools

In clinical practice, AI-driven decision support tools are increasingly used to help clinicians synthesize rapidly expanding trial and patient data. While terminology and evaluation technqiues may be novel, machine learning methods may not be too different from any other diagnostic or prognostic tool [16]. In this regard, clinical gestalt should be applied, as rigorous clinical validation against standardized metrics are still needed.

Several AI-enabled platforms designed specifically for oncologists have emerged in recent years. In 2025, the American Association of Clinical Oncology (ASCO) Guidelines Assistant program was launched for clinicians to query and navigate published guidelines [66]. Certain LLMs have also integrated oncology guidelines directly into their frameworks, such as the OpenEvidence partnership with the National Comprehensive Cancer Network (NCCN) by using adaptive decision trees that update over time through integration of new clinical data [67]. Notable innovations in AI, such as retrieval-augmented generation (RAG) architecture can allow individuals to “chat” with the NCCN guidelines, but this can predispose hallucinations or biases based upon how questions are perceived by the LLM [68]. Clinical decision support systems are also expected to be integrated into the electronic health records and can provide real-time decision support through an integrated recommended engine. This will require ongoing monitoring to avoid recycling of outdated clinical information.

At the individual patient level, machine learning algorithms may support clinical decision-making through prognostication based on multiomic and clinical variables. To give one example in resectable NSCLC, one machine learning-based survival prediction model demonstrated improved prognostic performance over conventional linear regression approaches that rely on TNM staging [69]. Several other machine learning survival models have been evaluated alongside traditional Cox regression, with the addition of SHaP (SHapley Additive exPlanations) methodology to enhance model interpretability [70]. SHaP is one innovative AI technique that quantifies the contribution of each input feature to an individual prediction, thereby improving transparency and facilitating clinical interpretation of otherwise “black box” predictive models [3]. While promising, SHaP is computationally expansive and may result in biased output, for example if two features assessed are interdependent.

### 3.3. AI Interventions in Thoracic Surgery and Radiation

In addition to informing treatment choices through prognostic models, AI-driven technologies can aid therapy strategies in both surgery and radiation planning. In thoracic surgery, AI-enabled platforms may integrate with robotic-assisted systems to enhance surgical precision, instrument control, and intraoperative decision-making. For example, three dimensional printed models of the lung and augmented reality (where images of the lung are displayed on top of the anatomy) have both shown to result in shorter operating time, less intraoperative blood loss, and shorter length of hospital stays [71]. While promising, three-dimensional printers do have inherent limiting factors such as precision, extensive preparation time, and high cost burden which impede real-world application. Newer cost-effective models with improved throughput are necessary before incorporation into clinical practice.

AI-assisted surgical video analytics have also demonstrated high accuracy in instrument recognition, anatomical structure identification, and operative phase recognition in cataract and laparoscopic gynecologic surgeries [72,73]. Similar video analytics could be integrated into thoracic surgeries; however, further technical validation and regulatory factors are warranted.

AI-based approaches have also demonstrated utility in radiation oncology treatment planning. Deep learning algorithms may, for example, automate tumor volumes and organs-at-risk delineation, tasks that traditionally require significant clinician time and are subject to interclinician variability [74,75]. These deep learning algorithms may also provide a benefit to individuals who may have had prior overlapping radiation fields.

AI innovations could also improve radiotherapy workflows, such as by synthesizing planning CT images from diagnostic imaging, rather than awaiting the formal simulation scan. One deep learning algorithm named deepPERFECT predicted planning CT with a non-inferior plan quality in a single-institution study [76]. A caveat to this study is that diagnostic CT scans can have significant changes in the relative spacial locations of tumors, which may lead to dosimetric inaccuracies and suboptimal dose constraints. Therefore, this planning technique may only be applicable to specific patients and would require a radiation oncologist to thoroughly examine the the spatial relationships between the tumor targets and nearby organs.

### 3.4. Treatment Selection and Toxicity Monitoring

Beyond local therapies, AI platforms can support precision treatment selection by predicting an individual’s response to systemic therapies. Machine learning models trained on genomic and clinical datasets have demonstrated the ability to predict patient-specific responses to targeted therapies [77]. AI approaches may also anticipate mechanisms of therapeutic resistance; for example, deep learning models applied to histopathology slides have been shown to predict gene mutation status (see Section 2.2) [78]. In addition to supporting standard-of-care therapies, AI can improve access to emerging therapies, such as via patient-trial matching algorithms for clinical trials [79].

AI models may also play a role in toxicity monitoring. A promising application for toxicity monitoring, currently in preclinical development, is the application of wearable devices that collect continuous biometric data, such as vitals, movement, and sleep patterns [80]. Integration of data streams from wearables with AI-based analytics has been explored for monitoring patient baseline function or treatment-related complications in both postoperative and systemic therapy settings [81].

One multicenter observational study in patients with metastatic NSCLC compared data obtained from the ActiGraph GT9X Link wearable device (including activity, sleep, and patient-reported symptoms recorded from a mobile application) with performance status assignment [81]. The trial identified moderate correlations between wearable-derived metrics and clinician-assessed performance status, suggesting a possible benefit to objective performance status assessment through wearable data.

Wearable devices have undergone several generational improvements since first reaching the commercial market, are reuseable, and have become increasingly affordable. While they may allow for accurate representations of lung cancer patients, many practical barriers to wearable implementation remain. This includes variable patient adherence, limitations in data integration with electronic health records, and inequalities in patient access to devices. Further multi-center prospective trials that implement wearable data to inform personalized treatment strategies or early detection of toxicity signals are warranted [80].

## 4. Ethical Integration of AI in Precision Oncology

The integration of AI in precision oncology requires careful consideration of clinical governance, regulatory, and ethical frameworks. In 2024, ASCO released principles for the responsible use of AI in clinical oncology (Table 3) [17]. The European Society for Medical Oncology (ESMO) has also issued guidance addressing the use of LLMs by patients, healthcare professionals, and healthcare institutions [82]. In addition, the World Health Organization (WHO) published guidance on the ethics of AI applications in healthcare at large [83]. All organizations emphasize the prioritization of human supervision and a human-centered approach to novel technology implementation.

In addition to ethical guidance, there continues to be need for harmonized standards for AI system evaluation and clinical deployment. Such standards can be informed by non-governmental bodies, such as the Friends of Cancer Research (see Section 2.2), and/or dictated by regulatory bodies, such as the FDA or European Medicines Agency (EMA) [84]. While we discussed select FDA-approved devices in the field of radiology (see Section 2.3), AI tools remain uncommonly implemented in routine clinical practice. As we expect the footprint of AI technologies to expand in coming years, transparent reporting, robust post-market surveillance, and harmonized regulatory frameworks will be even more central to ensure both safety and equitability of new technologies across patient populations.

Despite efforts by professional governance and national regulatory organizations, AI approaches remain vulnerable to bias and measurement errors, reflecting challenges long recognized in epidemiological and clinical research. Algorithmic bias may arise from limitations in datasets used for model training, including the underrepresentation or overrepresentation of certain clinical features, biologic parameters, management decisions, or patient outcomes [85]. Such imbalances may lead to models that perform unevenly across demographic groups or clinical settings. For example, in lung cancer, AI models developed primarily from datasets enriched for early-stage disease may underperform when applied to advanced-stage cases, potentially perpetuating disparities in diagnosis or treatment recommendations. The same case can be made for datasets enriched with actionable genomic alterations when applied to a general population.

Mitigation strategies for algorithmic bias include rigorous data quality assurance, deliberate efforts to improve dataset diversity, and ongoing model recalibration using updated real-world data [85]. Prior to tool development, defining a specific context of use for a technology is key. A model’s training dataset must incorporate multi-institutional data spanning diverse geographic regions, tumor subtypes, molecular landscapes, and demographic characteristics. The target population must match training data in the predefined context of use. In the post-marketing setting, employing ongoing machine learning techniques to identify and correct for performance disparities across patient subgroups will be pertinent.

At the same time, AI technologies may offer opportunities to promote equity in oncologic care. In resource-limited settings, such as in low- and middle-income countries, AI-enabled clinical decision support systems may augment the capacity of oncology clinicians by assisting in diagnostic interpretation, treatment planning, and clinical documentation [86]. However, there are potential risks: AI tools trained predominantly on datasets from high-income countries may perform poorly or cause harm when deployed in diverse global populations.

## 5. Conclusions

**Figure 1:**
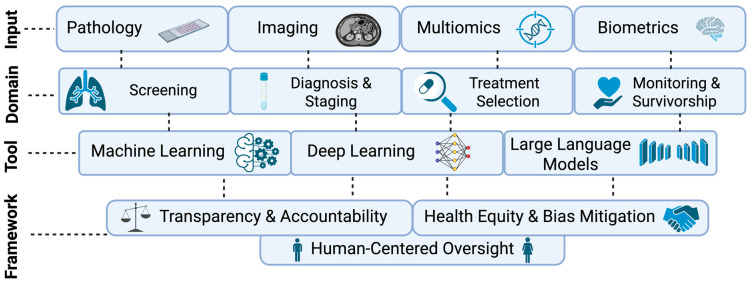
The artificial-intelligence-enabled precision lung cancer care continuum. AI tools have the potential to integrate patient-specific data inputs, such as pathology, imaging, multiomics, and biometrics. These data types can inform key clinical domains, including cancer screening, diagnosis and staging, treatment selection, and longitudinal toxicity and efficacy assessment during active treatment and survivorship. By applying AI techniques ranging from machine learning and deep learning algorithms to large language models, high-dimensional data can be leveraged to advance precision medicine. Governance frameworks underpinning this continuum emphasize transparency, accountability, bias mitigation, and health equity to support ethical and equitable implementation of novel technologies. in “Created in BioRender. Azenkot, T (2026). https://BioRender.com/spdz10p (accessed on 12 March 2026)“.

Precision oncology in lung cancer increasingly relies on the integration of complex clinical, imaging, pathologic, and molecular data to inform diagnosis and treatment. Given access to myriad databases for model training, the heterogeneity of molecular landscape, and the central role of thoracic imaging, lung cancer is uniquely positioned for the adoption of AI-based technologies. As illustrated in Figure 1, AI tools may synthesize high-dimensional, multimodal data used in thoracic malignancy diagnosis and disease monitoring. Effective models may translate these data into actionable insights across discrete clinical domains, from cancer detection to survivorship monitoring. When developed, implemented, and surveilled within robust governance frameworks, AI tools have the potential to enable more personalized therapeutic selection and longitudinal disease monitoring.

Across the continuum of lung cancer care, AI-driven tools are evolving from proof-of-concept models toward clinically deployable systems. In diagnostics, AI-based approaches such as pathomics and radiomics are expanding the information that can be derived from routine clinical data. These tools have potential to enable more efficient cancer screening, improved risk stratification, and less invasive approaches to tumor characterization. AI models are beginning to predict genomic alterations, immune phenotypes, and other molecular features directly from imaging and pathology data. As multiomic datasets expand, AI will likely play an increasingly important role in integrating available data to generate comprehensive understandings of lung cancer biology. Standardized metrics to evaluate AI-based diagnostics, harmonized across governance and regulatory bodies, will be critical to their successful implementation.

In therapeutics, AI has demonstrated potential to accelerate drug discovery and optimize treatment strategies. Machine learning approaches are under evaluation to identify novel therapeutic targets and predict drug–target interactions. These technologies may help address two central challenges in targeted therapy for lung cancer, as follows: the ability to target novel biomarkers and development of treatment for resistance. In parallel, AI-powered clinical decision support tools are emerging to assist clinicians in local treatment planning, improved navigation of evolving clinical guidelines, and prediction of patient-specific responses to therapy. Explainability and interpretability will be key features to enable novel tools to be integrated into clinical decision-making.

Despite promising advances, several challenges remain in the applications of AI in daily clinical practice. Model generalizability, dataset bias, regulatory oversight, and the need for prospective clinical validation remain critical considerations. Enduring questions surrounding transparency, accountability, and data governance underscore the importance of maintaining human oversight in AI-augmented healthcare. Looking ahead, the continued integration of AI in precision lung cancer care will depend on further collaborative efforts among clinicians, data scientists, regulatory bodies, and patient advocates.

## Figures and Tables

**Table 1:** Definition of artificial intelligence and select applications, as adapted from the Food and Drug Administration (FDA) Digital Health and Artificial Intelligence Glossary [3].

Tool	Definition
Artificial intelligence (AI)	A machine-based system that can, for a given set of human-defined objectives, make predictions, recommendations, or decisions.
Machine learning	Set of techniques that can be used to train AI algorithms to improve performance at a task based on data.
Deep learning	Set of techniques that train neural networks.
Neural network	Computational model composed of an input layer that receives data, one or more hidden layers that process and identify patterns in data, and an output layer that presents the final output.
Large language model	Type of AI model that can apply learned patterns to predict and generate natural language responses or conduct tasks, such as summarization.
Generative AI	Subset of AI models that emulate the structure and characteristics of input data to generate derived synthetic content.

**Table 2:** Clinical applications of artificial intelligence across the lung cancer care continuum.

Clinical Domain	Tool	Clinical Application	Potential Clinical Impact
Cancer screening	Computer-aided detection and diagnosis	Automated nodule detection; lung cancer risk models	Improved diagnostic accuracy, efficiency, and scalability
Diagnostic pathology and imaging	Pathomics; radiomics	Histologic and genomic prediction	More efficient, reproducible, and less invasive diagnostics
Surgery and radiation planning	Image segmentation models; augmented reality guidance	Surgical navigation and radiotherapy planning	Reduce planning timing; improve precision, reproducibility
Drug discovery	Quantitative structure–activity relationship modeling	Target identification; compound-target matching; resistance mechanism prediction	Accelerate drug development, prioritization of therapeutic candidates
Prognosis modeling and treatment selection	Survival and prediction models	Response to systemic therapy prediction; patient–trial matching	Individualize risk stratification, treatment selection; improve clinical trial access
Efficacy and toxicity monitoring	Digital health analytics; wearable-based monitoring; radiomics	Early detection of treatment effects, toxicities	Enable early intervention; improve safety

**Table 3:** Core principles for the implementation of artificial intelligence (AI) across select health governance organizations, including the American Association of Clinical Oncology (ASCO, 2025 [17]), the European Society for Medical Oncology (ESMO, 2025 [82]), and the World Health Organization (WHO, 2021 [83]).

ASCO [17]	ESMO [82]	WHO [83]
Transparency	Transparency	Ensure transparency, explainability, and intelligibility
Informed stakeholders	—	Protect human autonomy
Equity and fairness	Validation	Ensure inclusiveness and equity
Accountability	Continuous monitoring	Foster responsibility and accountability
Oversight and privacy	Privacy protection	Promote responsive and sustainable AI
Human-centered application	Human oversight	Promote human well-being and safety

## Data Availability

The original contributions presented in this study are included in the article. Further inquiries can be directed to the corresponding author.

## Abbreviations

The following abbreviations are used in this manuscript:

AI	Artificial intelligence
ASCO	American Association of Clinical Oncology
ATOM	Accelerating Therapeutics for Opportunities in Medicine
AUC	Area under the curve
CADe	Computer-aided detection
CADx	Computer-aided diagnosis
CANDLE	Cancer Distributed Learning Environment
CT	Computed tomography
ELIC	Early Lung Imaging Confederation
EMA	European Medicines Agency
ESMO	European Society for Medical Oncology
FDA	Food and Drug Administration
HER2	Human epidermal growth factor receptor 2
IASLC	International Association for the Study of Lung Cancer
LLM	Large language models
MoDaC	NCI Predictive Oncology Model and Data Clearinghouse
MRI	Magnetic resonance imaging
NCCN	National Comprehensive Cancer Network
NCI	National Cancer Institute
NMR-QCS	Normalized membrane ratio-quantitative continuous scoring
NSCLC	Non-small cell lung cancer
PD-L1	Programmed death-ligand 1
PET	Positron emission tomography
RAG	Retrieval-augmented generation
ROC	Receiver operating characteristic
SHaP	SHapley Additive exPlanations
TCGA	The Cancer Genome Atlas
TPS	Tumor Proportion Score
TROP2	Trophoblast cell surface antigen 2
WHO	World Health Organization
